# Inclusion of Camelina, Flax, and Sunflower Seeds in the Diets for Broiler Chickens: Apparent Digestibility of Nutrients, Growth Performance, Health Status, and Carcass and Meat Quality Traits

**DOI:** 10.3390/ani10020321

**Published:** 2020-02-18

**Authors:** Malwina Zając, Bożena Kiczorowska, Wioletta Samolińska, Renata Klebaniuk

**Affiliations:** Institute of Animal Nutrition and Bromatology, University of Life Sciences, Akademicka Street 13, 20-950 Lublin, Poland; zajac.malwina@wp.pl (M.Z.); wioletta.samolinska@up.lublin.pl (W.S.); renata.klebaniuk@up.lublin.pl (R.K.)

**Keywords:** oil seeds, productive parameters, blood parameters, chicken meat, nutrients

## Abstract

**Simple Summary:**

The presented results were obtained in a scientific trial, in which the addition of camelina, flax, and sunflower seeds to Ross 308 broiler diets was tested in terms of improvement of production efficiency, health status parameters, and quality of poultry meat. The results of the research showed that the tested full-fat seeds can be considered good dietary ingredients with a positive effect on the poultry production process and, consequently, on the dietary value of poultry meat.

**Abstract:**

The study determined the effect of the addition of 15% of camelina, flax, and sunflower seeds to iso-caloric and iso-nitrogenous diets for broiler chickens during 21–42 days of age on the nutrient digestibility, production traits, slaughter analysis parameters, hematological indices, blood mineral elements, and dietary value of breast and drumstick meat. Two hundred one-day-old broiler chickens were assigned to four groups (treatments) with five replicates (10 birds per cage, 5 females and 5 males). The experiment lasted 6 weeks. Broiler chickens receiving diets supplemented with camelina and flax seeds exhibited an increase (*p* < 0.05) in average body weight and a decrease (*p* < 0.05) in the ether extract content and energy digestibility of the diets. Moreover, the best carcass quality with a high proportion of muscles and low abdominal fat content (*p* < 0.05) was noted in broilers fed flax- and sunflower-enriched diets. The treatments with the oil seeds reduced the ether extract content and the calorific value of breast and drumstick muscles. The flax seeds contributed to an increase in the Fe content in drumstick muscles. Additionally, some blood parameters were influenced by the flax seed supplementation, e.g., the level of hemoglobin declined (*p* < 0.05) and the iron level in plasma increased (*p* < 0.05). It can be concluded that the camelina, flax, and sunflower seeds can be regarded as good dietary components with positive effects on the dietary value of poultry meat.

## 1. Introduction

The primary aim of animal production is to provide high nutritional and dietary value of meat, which mainly depends on its chemical composition. Currently, attention is paid not only to the content of essential nutrients but also to the content of minerals and biologically active substances [1,2]. The quality of final products, like meat quality, in large-scale poultry production is ensured mainly through appropriate optimization of the composition of poultry diets [3]. Addition of full-fat seeds into diets for broiler chickens can modify the content of nutrients in birds’ muscles to a certain extent, yielding a product with desired nutritional and dietary properties [4,5,6]. In this respect, the seeds of local oil-bearing plants, such as flax, camelina, or sunflower, seem to be extremely valuable [7,8,9].

The major focus in the literature on the supplementation of birds’ diets with flax, camelina, or sunflower seeds is placed on reduction of the ratio of n-6/n-3 fatty acids in poultry products [6,10,11,12]. Simultaneously, researchers report various effects of these seeds administered in diets on production efficiency. Rama Rao et al. [11] and Alagawany et al. [13] observed that administration of oil seeds contributed to higher body weight gains, reduced feed intake, and better slaughter analysis parameters.

Oilseeds also influence the digestive tract and digestion processes in poultry. This is connected with the relatively large amounts of dietary fiber in oilseeds, a significant part of which (ca. 1/3) dissolves in water, forming highly viscous solutions. The high viscosity of digesta may limit the digestion and absorption of nutrients in young birds [14,15,16]. Camelina, flax, and sunflower seeds also contain anti-nutritional components: linatin, cyanogenic glycosides, phytones, trypsin inhibitors, lignans, and saponins [17,18]. These factors can limit the potential of these seeds to be used in poultry production to a certain extent. Such doubts were raised by Ryhänen et al. [12] and Pekel et al. [19]. They reported a negative effect of oilseeds in poultry diets on production effects, especially when used in high doses. They noted impaired feed conversion and decreased feed intake during the starter period.

Given such diverse information on the potential nutritional application of camelina, flax, and sunflower seeds in poultry production, it seems advisable to conduct further research in this field. Moreover, there is little information in the literature on the effect of seeds administered in feed mixes to chickens on the content of macronutrients and micronutrients in their meat.

Therefore, the aim of the study was to analyze the impact of the addition of raw full-fat camelina, flax, and sunflower seeds to diets for broiler chickens on the basic production parameters, nutrient digestibility, hematological profile, and the nutritional and dietary quality of meat and the basic nutrient and mineral profile.

## 2. Materials and Methods

### 2.1. Oil Seeds, Experimental Design, and Management

Full-fat unprocessed camelina (*Camelina sativa* L. Crantz) Luna cv., flax (*Linum* L.) Opal cv., and dehulled sunflower (*Helianthus* L.) Lech cv. were purchased in a specialist store (Seed Centre, Lublin, Poland) as a locally produced certified plant material originating from crop harvesting in 2017. The oilseeds were grown in areas located in southern and eastern Poland. The chemical composition of the seeds is shown in Table 1. Oilseeds from three different batches purchased at different dates were subjected to chemical analysis. Three random samples were collected from each batch of seeds and analyzed in triplicate. The content of dry matter and basic nutrients in the ground oilseed samples (250 g seeds/variety) was determined according to standard AOAC procedures [20] described in Section 2.4.1.

The experiment was carried out with the permission from the Second Local Ethics Committee at the University of Life Sciences in Lublin (No. 35/2015) on a poultry farm (Sędziszów, Malopolskie province, Poland). One-day-old broiler chickens Ross 308 (200 birds) were randomly assigned to 4 dietary treatments with 5 replicate cages per treatment (5 males and 5 females/cage). The body weight of the 1-day-old broiler chickens was 42.6 ± 0.1 g. The experiment was carried out for 6 weeks. The broiler chickens were reared in 1-m^2^ cages. The birds were provided with continuous access to feed and water. The lighting scheme controlled the daylight schedule, temperature, and humidity of the air in the poultry house [21]. Vaccination and vet care treatment were provided according to the guidelines for broiler chickens specified by Aviagen [21].

The experimental feed diets were based on cereal meal middlings (wheat and corn) and post-extraction soybean meal (Table 2). Three types of mixtures, starter (0 to 21 days), grower (21 to 35 days), and finisher (35 to 42 days), were used in the experiment. The starter diet was administered in the crumble form while the grower and finisher diets were granulated. From rearing day 22, the broilers were fed according to the methodological design, with 15% of oilseeds in the diets as an experimental factor. The experimental mixtures were iso-energetic and iso-nitrogenous and balanced in accordance with feeding recommendations for broiler chickens [22].

### 2.2. Growth Performance and Apparent Digestibility of Nutrients

For each cage, the body weight (BW) of the broiler chickens and the average daily feed intake (ADFI) were recorded at 21, 35, and 42 days of life. The body weight gains (BWGs) and feed conversion ratio (FCR) were calculated in the grower and finisher periods. The mortality rates were recorded daily. The weight of dead broiler chickens was included to calculate the average weight gain, feed intake, and FCR.

Feed digestibility was evaluated using the indicator method (with acid-insoluble ash as an internal marker) [23]. The analysis of nutrient digestibility was carried out from rearing day 22 to 28 in the grower mixture and from rearing day 36 to 42 in the finisher mixture. The birds were acclimated to the feed for 4 days. Their droppings were collected for the next 3 days. Each mixture was assessed by examination of four broiler chickens selected randomly from each cage at the final grower and finisher stage. During the digestibility analyses, the amount of feed consumed and the amount of droppings were determined. Collected feces were dried at 60 °C and weighed and subjected to determination of the dry matter content (Method 925.09), crude ash (Method 923.03), ether extract (Method 920.39), and organic matter [20]. The birds were kept in conditions specified in the experimental design (Section 2.1). The content of dry matter and organic matter was determined in collected droppings [20]. The content of nitrogen was determined according to Krogdahl and Dalsgard [24]. The nutrient digestibility coefficient and the content of nitrogen-corrected metabolizable energy (MEn) were calculated for experimental mixtures as recommended in the European Table of Energy Values for Poultry Feedstuffs [25].

One female and one male broiler chicken with the body weight close to the average value were selected from each cage for dissection. Selected chickens were not given feed 10 h before slaughter, whereas constant access to water was ensured. The slaughter was carried out by decapitation. After the slaughter, a simplified dissection analysis was performed. Liver, gizzard, heart, breast, and drumstick muscles, and abdominal fat were collected. The tissues were weighed, packed in specially marked bags, and frozen at −25 °C until chemical analyses [26].

### 2.3. Hematological and Blood Mineral Analysis

The chickens selected for the slaughter were also assigned for blood sampling (two broiler chickens/cage). The blood sampling and hematological analyses of red blood cells (RBCs), packed cell volume (PCV), hemoglobin (HGB), mean cell volume (MCV), mean cell hemoglobin (MCH), and mean corpuscular hemoglobin concentration (MCHC) were described in a previous article [27]. The contents of calcium, magnesium, phosphorus, copper, iron, and zinc were determined in plasma without hemolysis sign using methods described in a previous article [28].

### 2.4. Sample Collection and Chemical Analyses

#### 2.4.1. Oilseeds and Diets

The content of basic nutrients, dry matter (Method 925.09), crude ash (Method 923.03), crude protein (Method 920.87), ether extract (Method 920.39), and crude fiber (Method 962.09), in the oil seeds and diets was determined by AOAC [20], and the MEn content was calculated [25].

Fat was extracted from the seeds using the diethyl ether solvent with the Soxhlet extraction method (Method 920.39) [20]. Gas chromatography was used for determination of the fatty acid composition. The procedure was carried out after conversion of the fats to fatty acid methyl esters (FAME) [29]. The technical and chemical details of the method used for determination of fatty acids are described in an earlier publication [9]. Fatty acids were expressed as a percentage of the total fatty acids identified [30].

Tocopherols were chemically determined in the oilseeds by extracting 0.5 g of the sample with 5 mL hexane containing 20 mg/L DYN [31].

The details of the method for determination of xanthophylls and total phenolic content in oilseeds were presented in an earlier publication by Kiczorowska et al. [9].

The amino acid contents in the experimental diets were determined using an automatic amino acid analyzer (AAA 400; Ingos, Prague, Czech Republic) after previous acid hydrolysis with 6 M HCl (method 994.12) [20]. Cysteine and methionine were determined after oxidative hydrolysis [32].

The contents of Ca, Mg, P, Cu, Fe, and Zn in the seeds as well as Ca, Mg, and P in the diets were measured (three replicates of each sample) using flame atomic absorption spectrophotometry (FAAS) (Unicam 939/959AA-6300, Shimadzu Corp., Tokyo, Japan). Calcium was determined at λ = 422.7 nm, magnesium at λ = 285.2 nm, copper at λ = 324.8 nm, iron at λ = 248.3 nm, and zinc at λ = 213.9 nm [33]. Total P content was determined colorimetrically [34]. The individual stages of chemical determinations were described in previous articles [1,2].

#### 2.4.2. Breast and Drumstick Muscle of Broiler Chickens

The contents of moisture (Method 925.09), crude ash (Method 923.03), crude protein (Method 920.87), and ether extract (Method 920.39) were determined in breast and drumstick muscles [20]. The energy value of the analyzed breast and drumstick muscles is based on the Atwater general factors for the energy density of fat, protein, and carbohydrate (9, 4, and 4 kcal/g, respectively). In accordance with the regulation of the European Parliament and the EU Council of October 25, 2011, the value of the coefficient was adjusted to the calorific value of fiber, i.e., 2 kcal/g [35]. The energy value of the tested muscles expressed in kcal was converted to kJ using a coefficient of 4.1868.

The contents of Ca, Mg, P, Cu, Fe, and Zn in the chicken muscles were determined using flame atomic absorption spectrophotometry (FAAS) as described in Section 2.4.1. Chemical analyses were carried out using minerals determined in the chicken meat Standard Reference Material NCS ZC73016.

All analyses were performed in triplicate and all data were expressed as means.

### 2.5. Statistical Analysis

The data were analyzed using one-way analysis of variance (ANOVA; α = 95; *p* < 0.05). Statistica software (version 13.3; StatSoft, Tulsa, OK, USA) was used for the calculation of the mean values and the SEM. The cage was used as a statistical unit. The normality of data and homogeneity of variances were tested using the Shapiro–Wilk and Brown–Forsythe tests, respectively. Significant differences between the means were determined by the Tukey’s honestly significant difference (HSD) post hoc test:

Y_ij_ = μ + a_i_ + e_ij_,

where Y_ij_ is the measured variable, μ is an overall mean, a_i_—the dietary inclusion of the full-fat oilseeds (treatment), and e_ij_—the random error.

Pearson’s correlation coefficients (r) were used to determine the direction and intensity of the relationships between the basic nutrients and minerals in the oilseeds (camelina, flax, sunflower) and broiler chicken muscles (breast, drumstick).

## 3. Results

### 3.1. Productivity Parameters and Nutrient Digestibility

The basic productivity parameters of the broiler chickens, i.e., body weight (BW), average daily feed intake (ADFI), body weight gains (BWG), and feed conversion ratio (FCR), observed in the first rearing period did not differ between the groups (Table 3). The addition of 15% of oilseeds to the diets improved the efficiency of broiler chicken rearing. A particularly beneficial effect, i.e., high body weight (BW) in the finisher period (*p* = 0.038), was achieved through the supplementation with the camelina and flax seeds. Another positive effect, i.e., an average 5% reduction (*p* = 0.042) of the feed intake (ADFI) compared with the control, was noted after administration of the camelina and sunflower seeds in the diets. An equally important achievement of the use of oilseeds was the decline (*p* = 0.026) in chicken falls throughout the grower and finisher periods in comparison with the control treatment. The addition of the oilseed blends (*p* < 0.05) also improved the slaughter analysis parameters. Bird carcasses from the full-fat oilseed treatments were characterized by higher breast muscle content (*p* = 0.037) and greater gizzard weight (*p* = 0.044) as well as a smaller proportion of abdominal fat (*p* = 0.028), compared with the control treatment. The analysis of both the grower and finisher periods (day 21–42) in the nutrition experiment showed that the basic production parameters of the broiler chickens fed with mixtures containing the tested oilseeds did not differ significantly. Only the feed conversion ratio (FCR) value in experimental groups supplemented with camelina and flax seed mixtures was significantly higher than in the control group (*p* = 0.035).

The present study also consisted of an analysis of the digestibility of basic nutrients (Table 4). The 15% addition of full-fat seeds contributed to a reduction of the digestibility of ether extract in the feed in both the grower (21–35 days, *p* = 0.038) and finisher periods (36–42 days, *p* = 0.026), compared to the control group. The lowest digestibility of fat, even by 14% compared to the control treatment, was detected in the CAM broiler chickens. All chickens from the full-fat seed treatments were also reported to have reduced gross energy digestibility (*p* = 0.033) in the grower period. The digestibility of the other nutrients in the feed did not differ significantly between the full-fat seed treatments and the control treatment.

### 3.2. Basic Nutrients and Mineral Elements in Broiler Chicken Muscles

The addition of the oilseeds to the broiler chicken diet reduced the ether extract content on average by 21% in breast muscles (*p* = 0.024) and approximately 34% in drumstick muscles (*p* = 0.037), in comparison with the control (Table 5). The drumstick muscle of the experimental chickens was also characterized by a ca. 15% lower (*p* = 0.042) calorific value in comparison with the muscles of chickens fed the standard diets. The largest decrease in the fat content and calorific value was recorded in the muscles of chickens fed with the addition of the flax and sunflower seeds. Additionally, the content of crude ash was lower (*p* = 0.038) in the breast muscles of the CAM, FLA, and SUN chickens than in the control. No significant differences were found in the content of the other nutrients in the poultry muscles.

The addition of the flax seeds into the diets for broiler chickens increased (*p* = 0.038) the content of Fe in drumstick muscle by approximately 11%, compared with the control group (Table 5).

The values of the correlation coefficients (r) between basic nutrients and some minerals in the oilseeds as well as the breast and drumstick muscles of chicken broilers supplemented with CAM, FLA, and SUN are presented in Table 6.

There were mainly strong correlations between the contents of the basic nutrients and elements in the seeds and in the breast and drumstick muscles. High positive and negative correlations (r > 0.6) were observed mainly between the nutrient content in the chicken muscles and camelina seeds: Ca, Mg, P, Cu, Fe, Zn, and fat, in the chicken muscles and flax seeds: Ca, Mg, P, Cu, Fe, Zn, fat, and crude ash, and in the chicken muscles and sunflower seeds: Ca, Mg, P, Cu, Fe, Zn, and fat (*p* < 0.05).

The values and trends of the correlation coefficients obtained for the components are probably associated with their physicochemical properties, antagonistic or synergistic interactions, bioavailability, as well as coexistence and co-involvement with other components in physiological and metabolic processes.

### 3.3. Hematological Indices and Blood Minerals in Broiler Chickens

In the experiment, selected hematological indices were determined to evaluate the health status of the birds (Table 7). The CAM, FLA, and SUN treatments did not induce changes in the levels of such indices as RBC, MCHC, MCH, MCV, and PCV. In contrast, the dietary inclusion of flax seeds to the diet decreased the hemoglobin level in comparison with the control (by 9%) and the other treatments (*p* = 0.040).

The present study showed an influence of FLA on the iron content in the blood plasma of the broiler chickens (*p* < 0.001). The addition of the flax seeds to the diet increased the plasma iron concentration in the broiler chickens, compared with the control, CAM, and SUN treatments (Table 7).

There was a higher plasma level of magnesium in the FLA treatment than in CAM (*p* = 0.048), but the differences between the other treatments were not statistically significant.

## 4. Discussion

The inclusion of 15% of full-fat camelina, flax, and sunflower seeds to the experimental diets for broilers at 21–42 days of age resulted in an improvement of some production parameters, e.g., body weight in the finisher period of rearing, average daily feed intake, weight of breast and drumstick muscles and gizzard. This phenomenon is very interesting, especially given the significantly lower fat digestibility noted in chickens fed the experimental camelina- and flax-supplemented diets. When energy- and protein-balanced diets are provided to animals, it is difficult to obtain varied production effects expressed in basic parameters, such as body weight or daily feed intake. However, the extremely healthy fat composition of oilseeds, i.e., camelina, flax, and sunflower seeds, can probably stimulate the bird’s organism to intensified growth and development of tissues and organs [36]. A positive effect of the use of camelina seeds in the nutrition of broiler chickens was noted by Ciurescu et al. [3] in their investigations of broiler chickens. The highest body weight gains were recorded in birds receiving mixtures with 5% camelina addition, but the most effective reduction in the feed intake and the best slaughter parameters were observed in chickens receiving these seeds at a dose increased to 10%. In contrast, Aziza et al. [37] administered 2.5%, 5%, and 10% of camelina meal to broiler chickens and reported no differences in the body weight gain or feed efficiency, compared with the control. In turn, as reported by Gonzalez and Leeson [14], already a 20% dose of camelina in the diet can induce negative production effects. In the present study, the flax seeds proved to be equally beneficial in the experiments. Similarly, Apperson and Cherian [6] reported a multidirectional positive effect of 10% and 15% addition of flax seeds to diets on the gastrointestinal health status in broiler chicken, e.g., improved intestinal morphometric parameters and reduced viscosity of feces. However, they did not detect significant differences in the average body weight, average daily gain, or feed consumption. Similarly, Konieczka et al. [10] did not observe a significant effect of the use of flax seeds in broiler chicken nutrition on rearing parameters. However, they reported a beneficial impact of this additive on the quality of poultry meat and improvement of slaughter analysis parameters accompanied by a significant decrease in the content of abdominal fat in carcass. As demonstrated by Waititu et al. [38] and Attia et al. [15,39], sunflower meal can replace up to 50% of soybean meal without depressing growth performance in either the starter or the finisher period. The authors suggest that the inclusion of sunflower meal in broiler diets does not affect productivity or barn hygiene management. Slightly different results were presented by Amerah et al. [40], who supplemented broiler chicken diets with 5–6%, 8–10%, and 8–12% of sunflower meal. They observed no significant differences in the average body weight, feed consumption, feed efficiency mortality, and organ weight.

In the present study, the fat digestibility and energy parameters were reduced. This phenomenon was especially intensified in the groups of broiler chickens fed mixtures containing camelina and flax seed. The reduction in the efficiency of digestion of oilseed fat in the chicken intestine is associated with the high content of non-starch polysaccharides (NSPs). Flax seeds contain water-soluble and insoluble NSP forms. In these seeds, over 46% of NSP are represented by the water-soluble fraction [41]. As reported by Rebol’e et al. [42] and Alzueta et al. [43], the high levels of water-soluble NSP in diets for chickens are associated with increased digesta viscosity and decreased digestibility of all nutrients, particularly fat [40]. Whole flax seeds also contain other anti-nutritional substances, such as linamarin, mucilages, or cyanogenic glycosides, which can exert a negative effect on nutrient digestion [10,40]. Young birds are particularly sensitive to these factors; therefore, high doses of oilseeds are not recommended in the first period of rearing [6,37]. In these studies, these concerns were a basis for inclusion of full-fat oilseeds in broiler chicken diets only in the second and third rearing periods. Rodríguez et al. [44] analyzed the effect of diets for male broilers containing 0, 80, 120, and 160 g/kg of flaxseed on the digestibility of nutrients. The authors observed a decrease in nitrogen retention and digestibility of amino acids, ether extract, and fatty acids in diets with an increasing share of flax seed. The viscosity of jejunal digesta was markedly increased by each increment of linseed in the diets. This is attributable to the presence of mucilage in linseed. Jia and Slominski [41] used carbohydrase enzymes to increase the digestibility of flax seeds. Similar studies were conducted by Apperson and Cherian [6], who reported increased digestibility of not only fat but also ALA and n-3 fatty acids. The enzyme supplementation also influenced the expression of genes involved in lipid metabolism.

It was found in the present study that the inclusion of oilseeds into broiler chicken feed mixtures reduced the fat content in their muscles (breast and drumstick). This may have been caused by the reduced digestibility of fat in the diet. The lower fat accumulation in the muscles was also observed by Rebolé et al. [42] in the muscles of chickens fed with high doses of sunflower in the diet (10%, 15%, and 20%). They explained this phenomenon by an increase in the lipid oxidation rate and lower synthesis of endogenous fatty acid. As suggested by Sanz et al. [45], the metabolic utilization of energy from fat in broiler chickens is determined by fatty acid saturation. The authors propose that circulating fat can be absorbed by muscle tissue and used as a direct source of energy rather than stored in adipose tissue. There are also other literature reports on the effect of flax in diets for poultry on the chemical composition of meat. No significant effect on the fat content in muscles was observed by Jankowski et al. [46] in their research on turkeys fed a diet of linseed oil and by Rahimi et al. [47] in investigations of broilers fed flax seeds. However, the authors emphasize the beneficial dietary modifications of fat quality, i.e., a significant increase in the concentrations of n-3 polyunsaturated fatty acids (PUFAs). Similarly, in the present study, a beneficial effect of the nutritional use of oilseeds on the dietary properties of poultry meat was found. The breast and drumstick muscles of broiler chickens fed with the mixtures containing camelina, flax, and sunflower seeds exhibited significantly lower fat and energy contents than the control birds. Reduction of the calorie content in meat is desirable in terms of the proper composition of a human diet and is being increasingly expected by consumers.

There is no information in the literature about the impact of nutrition supplemented with full-fat seeds on the mineral composition in chicken muscles. The higher Fe concentrations detected in the muscles of the FLA broiler chickens can be associated with the high blood level of the element, which to some extent reflects its concentration in the organism. The level of iron in the body largely depends on the diet and gastrointestinal absorption [48]. In the present study, the content of the other minerals in the breast and drumstick muscles did not differ significantly, regardless of the energy source in the diet (camelina, flax, and sunflower seeds).

The present study also involved analysis of the relationships between basic nutrients and mineral elements in the oilseeds in mixtures for broilers and their breast and drumstick muscles. The values and trends of the correlation coefficients obtained for the components are probably associated with their physicochemical properties, antagonistic or synergistic interactions, bioavailability, as well as coexistence and co-involvement in metabolic and physiological processes with other ingredients.

Hematological indices are useful in assessment of the potential toxicity of feed, especially in the case of feed components that can affect blood parameters and animal health. The values of the analyzed blood indicators corresponded to the reference values specified for this species [49,50]. Similarly, other authors reported a negative effect of the addition of flax seeds into the diet on the hematological indicators in animals. Investigations consisting in the supplementation of diets for rabbits with two flaxseed varieties demonstrated a decline in the values of RBC, HgB, MPV, and PCV in the blood of the animals [51]. Rajesha et al. [52] reported reduced PCV and RBC levels in the blood of hens correlated with the level of flax seeds in the diet. However, they did not observe an effect of the flax seeds on the HgB level.

In the present study, the reduction in the HgB level induced by the FLA treatment may have been associated with the content of cyanogenic glycosides (linamarins, linustatins, and neolinustatin) and enzymes (β-bis-glucosidase, β-monoglucosidase, and α-hydroxynitrile lyase) involved in the hydrolysis of cyanogenic glycosides and release of hydrocyanic acid [53]. Hydrogen cyanide is toxic to animal organisms, as it contributes to inhibition of cytochrome oxidase activity in the mitochondrial respiratory chain. It is also an effective inhibitor of other enzymes due to its ability to bind with iron, manganese, or copper ions, which are functional groups of many enzymes. Hydrogen cyanide can also bind to hemoglobin to form cyanohemoglobin, which does not dissociate to HgB [54,55].

Blood serum and plasma often serve as a biological matrix for assessment of the proper supply of elements and their transformations in the organism. Their content in the body is a result of a dynamic balance of the demand, supplied quantity, and availability [56,57]. The concentration of iron in blood depends on, e.g., its content in the diet, gastrointestinal absorption, and the intensity of hemoglobin breakdown and synthesis [48]. Probably, the presence of cyanogenic glycosides in flax seeds and their effect on the formation of cyanomethemoglobin mobilizes iron stores, increasing its level in the blood plasma.

Oilseeds contain a substantial amount of minerals, in particular phosphorus, magnesium, iron, zinc, and calcium, which, however, are characterized by reduced bioavailability. These seeds also contain substances with anti-nutritional properties, which may exert a negative effect on the organism, e.g., by limitation of the absorption of nutrients or even toxic effects. The main anti-nutrients found in oilseeds are phytic acid, tannins, cyanogenic glycosides in flax seeds, as well as glucosinolates and sinapine mainly in seeds of *Brassicaceae* plants [58,59]. The available literature presents the results of numerous nutritional studies suggesting poor bioavailability of such minerals as phosphorus, zinc, calcium, magnesium, or even iron from phytic acid-rich diets [60,61]. The content of phytic acid in the oilseeds was not analyzed in the present study. However, there are many reports confirming considerable amounts of this compound, especially in camelina seeds [58,62], which may explain the low plasma magnesium level in the broiler chickens fed the diet with camelina seeds.

## 5. Conclusions

The positive effect of the use of the 15% camelina, flax, and sunflower seed addition in the diets at 21–42 days of age improved the average body weight, average daily feed intake (in the finisher period), and some slaughter parameters in the broiler chicken. The inclusion of oilseeds into the mixture for broiler chickens, especially camelina and flax, had a positive effect on the dietary and nutritional value of chicken muscles. The negative effect of the flax seed diet was reflected in the weakened fat digestibility. An equally disturbing effect was the decrease in the hemoglobin blood content in chickens fed the flax diet. Based on the production results obtained so far, camelina, flax, and sunflower seeds seem to be good diet components as they exert positive effects on the dietary value of poultry meat. However, there is still a need for research to elucidate the mechanisms associated with the ability of oilseeds to potentiate the retention of elements and their impact on the health status of birds.

## Figures and Tables

**Table 1:** Chemical composition of camelina, sunflower (dehulled), and flax seeds.

Compounds	Camelina	Sunflower	Flax
Basic nutrients, g/kg dry matter			
Dry matter	863	851	915
Crude ash	42.8	34.6	37.8
Crude protein ^1^	248	239	206
Ether extract	394	492	448
Crude fiber	115	46.3	79.4
Fatty acids, % of total FAME			
C16	5.38	5.67	5.18
C18	2.56	3.74	4.28
C16:1	0.110	0.091	0.080
C18:1	14.8	27.2	18.2
C18:2	17.6	61.5	13.6
C18:3	35.8	0.113	57.8
SFA ^2^	11.2	11.0	9.87
MUFA ^3^	33.4	27.5	18.5
PUFA ^4^	55.5	61.4	71.7
Minerals, mg/100 g fresh matter			
Ca	370	285	110
P	745	670	780
Mg	405.3	318	354
Cu	1.20	0.73	1.42
Fe	14.5	8.53	5.34
Zn	7.08	4.82	5.24
Bioactive components, total in fresh matter ^5^			
Tocopherols, μg/g	410	149	318.4
Xanthophyll, μg/g	41.3	35.8	24.5
Total phenolic compounds, mg/100 g	880	2245	85.7

Results are the average of 9 samples in three replicates, ^1^ Calculated by Kjeldhal nitrogen N × 6.25, ^2^ SFA—Saturated fatty acid, ^3^ MUFA—Monounsaturated fatty acids, ^4^ PUFA—Polyunsaturated fatty acids, ^5^ Fresh sample.

**Table 2:** Ingredients and chemical composition (g/kg, as a fed basis) of experimental diets.

Item	Diets ^1^											
	Starter (0 to 21 Days)				Grower (21 to 35 Days)				Finisher (35 to 42 Days)			
	Control	CAM	FLA	SUN	Control	CAM	FLA	SUN	Control	CAM	FLA	SUN
Diet composition, g/kg												
Wheat	200.0	200.0	200.0	200.0	230.0	230.0	230.0	230.0	270.0	270.0	270.0	270.0
Soybean meal, 46% crude Protein	394.4	394.4	394.4	394.4	359.3	279.3	299.3	294.3	312.5	242.5	262.5	257.5
Maize	300.0	300.0	300.0	300.0	300.0	300.0	280.0	285.0	300.0	300.0	280.0	285.0
Soybean oil	60.0	60.0	60.0	60.0	70.0				80.0			
Camelina seeds						150.0				150.0		
Flax seeds							150.0				150.0	
Sunflower seeds, dehulled								150.0				150.0
Dicalcium phosphate	18.3	18.3	18.3	18.3	18.0	18.0	18.0	18.0	18.0	18.0	18.0	18.0
Limestone	12.0	12.0	12.0	12.0	10.0	10.0	10.0	10.0	7.0	7.0	7.0	7.0
NaCl	3.3	3.3	3.3	3.3	3.3	3.3	3.3	3.3	3.3	3.3	3.3	3.3
DL-Met ^2^	3.6	3.6	3.6	3.6	3.3	3.3	3.3	3.3	3.3	3.3	3.3	3.3
L-Lys ^3^	3.4	3.4	3.4	3.4	3.6	3.6	3.6	3.6	3.4	3.4	3.4	3.4
Vitamin-mineral premix ^4^	5.0	5.0	5.0	5.0	2.5	2.5	2.5	2.5	2.5	2.5	2.5	2.5
Chemical composition, g/kg												
MEn (MJ/kg) ^5^	12.5	12.5	12.5	12.5	13.0	13.0	13.0	13.1	13.3	13.2	13.3	13.3
Crude protein	221	221	221	221	209	205	204	209	192	196	195	198
Lys	14.3	14.3	14.3	14.3	12.8	12.8	12.5	13.02	11.6	11.1	11.7	11.6
Met + Cys	10.5	10.5	10.5	10.5	9.68	9.73	9.29	9.81	8.94	8.77	8.63	9.08
Ca	9.84	9.84	9.84	9.84	8.87	8.57	8.25	8.73	7.86	7.88	7.98	8.01
P	6.61	6.61	6.61	6.61	6.29	6.69	6.56	6.38	6.21	6.58	6.62	6.64
Mg	0.612	0.612	0.612	0.612	0.383	0.432	0.411	0.392	0.394	0.450	0.432	0.410

^1^ Control—diet without oil seeds, CAM—diet with 15% camelina seeds, SUN—diet with 15% sunflower seeds, FLA—with 15% flax seeds; ^2^ Evonik Degussa Gmbh. Essen. Germany (per kilogram of 990 g methionine); ^3^ Ajinomoto Eurolysine S.A.S. Amiens. France (per kilogram of 780 g lysine); ^4^ Added minerals and vitamins per kg of starter diet: Mn, 100 mg; I, 1 mg; Fe, 40 mg; Zn, 100 mg; Se, 0.15 mg; Cu, 10 mg; vitamin A, 15,000 IU; vitamin D3, 5000 UI; vitamin E, 75 mg; vitamin K3, 4 mg; vitamin B1, 3 mg; vitamin B2, 8 mg; vitamin B6, 5 mg; vitamin B12, 0.016 mg; biotin, 0.2 mg; folic acid, 2 mg; nicotic acid, 60 mg; pantothenic acid, 18 mg; choline, 1800 mg. Added minerals and vitamins per kg of grower diet: Mn, 100 mg; I, 1 mg; Fe, 40 mg; Zn, 100 mg; Se, 0.15 mg; Cu, 10 mg; vitamin A, 12,000 IU; vitamin D3, 5000 UI; vitamin E, 50 mg; vitamin K3, 3 mg; vitamin B1, 2 mg; vitamin B2, 6 mg; vitamin B6, 4 mg; vitamin B12, 0.016 mg; biotin, 0.2 mg; folic acid, 1.75 mg; nicotic acid, 60 mg; pantothenic acid, 18 mg; choline, 1600 mg. Added minerals and vitamins per kg of finisher diet: Mn, 100 mg; I, 1 mg; Fe, 40 mg; Zn, 100 mg; Se, 0.15 mg; Cu, 10 mg; vitamin A, 12,000 IU; vitamin D3, 5000 UI; vitamin E, 50 mg; vitamin K3, 2 mg; vitamin B1, 2 mg; vitamin B2, 5 mg; vitamin B6, 3 mg; vitamin B12, 0.011 mg; biotin, 0.05 mg; folic acid, 1.5 mg; nicotic acid, 35 mg; pantothenic acid, 18 mg; choline, 1600 mg; ^5^ MEn = metabolizable energy in the mixtures corrected to zero nitrogen balance (MEn, MJ/kg = 0.1551 × % crude protein + 0.3431 × % crude fat + 0.1669 × % starch + 0.1301 × % total sugar [expressed as sucrose]).

**Table 3:** Productive performance, mortality, and carcass traits of broilers chickens fed a control, camelina, flax, and sunflower diet ^1^.

Items	Treatments ^2^				Statistical Parameters	
	Control	CAM	FLA	SUN	SEM ^3^	*p*-Value ^4^
Productivity Parameters						
1–20 day (starter period)						
BW ^5^, g	639	652	656	644	53.2	0.145
ADFI ^6^, g/day	58.3	59.1	57.9	58.2	33.4	0.089
BWG ^7^, g/chicken	678	694	695	683	2.04	0.209
FCR ^8^, kg/kg	1.67	1.68	1.69	1.65	0.05	0.203
21–35 day (grower period)						
BW ^5^, g	1713	1814	1794	1783	63.1	0.126
ADFI ^6^, g/day	1102	1103	1084	1056	47.2	0.157
BWG ^7^, g/chicken	1265	1304	1315	1289	3.15	0.241
FCR ^8^, kg/kg	2.04	2.01	1.95	1.94	0.03	0.089
36–42 day (finisher period)						
BW ^5^, g	2415 ^b^	2693 ^a^	2617 ^a^	2565 ^a,b^	78.6	0.038
ADFI ^6^. g/day	1402 ^a^	1311 ^b^	1355 ^a,b^	1330 ^b^	97.3	0.042
BWG ^7^, g/chicken	543	539	542	551	3.14	0.127
FCR ^8^, kg/kg	1.96	1.86	1.82	1.87	0.07	0.138
21–42 day (grower and finisher period)						
BW ^5^, g	2064	2254	2206	2174	95.30	0.082
ADFI ^6^, g/day	1252	1207	1220	1193	87.20	0.145
BWG ^7^, g/chicken	904	922	929	920	5.15	0.484
FCR ^8^, kg/kg	2.00 ^a^	1.86 ^b^	1.89 ^a,b^	1.91 ^a^	0.15	0.035
Mortality, %	3.12 ^a^	2.14 ^b^	2.15 ^b^	1.57 ^c^	0.04	0.026
Slaughter parameters						
Dressing percentage, g/kg	76.1	78.2	78.6	76.9	3.45	0.241
Abdominal fat, g	18.1 ^a^	7.46 ^d^	9.51 ^c^	14.3 ^b^	0.18	0.028
Muscles weight, g						
Breast muscle, g	348 ^b^	359 ^b^	401 ^a^	378 ^a^	2.41	0.035
Drumstick muscle, g	152 ^a^	149 ^a,b^	156 ^a^	137 ^b^	9.84	0.016
Organ weight, g						
Liver	53.6	47.8	48.4	54.6	0.57	0.135
Stomach	29.3 ^b^	34.4 ^a^	32.1 ^a,b^	36.8 ^a^	0.33	0.044
Heart	12.6	13.1	11.8	12.2	0.09	0.257

^1^ Data represent the mean of 5 cages (10 broiler chicken/cage) per treatment; ^2^ Control—diet without oilseeds; CAM—diet with 15% camelina seeds, FLA—with 15% flax seeds; SUN—diet with 15% sunflower seeds; ^3^ SEM—the standard error of the mean, ^4^ *p* < 0.05—statistical differences; ^a,b,c^—statistical differences; ^5^ BW—average body weight; ^6^ ADFI—average daily feed intake; ^7^ BWG—body weight gain; ^8^ FCR—feed conversion ratio.

**Table 4:** Apparent nutrients and energy digestibility of broilers chickens fed a control, camelina, flax, and sunflower diet, % ^1^.

Items	Treatments ^2^				Statistical Parameters	
	Control	CAM	FLA	SUN	SEM ^3^	*p*-Value ^4^
21–35 day (grower)						
Dry matter	75.4	81.5	82.1	79.8	0.45	0.239
Crude protein	74.5	78.4	75.6	75.9	0.67	0.324
Ether extract	73.8 ^a^	63.4 ^b^	65.6 ^b^	70.9 ^a,b^	0.38	0.038
Organic matter	80.6	84.8	85.3	86.7	0.51	0.127
Gross energy	87.6 ^a^	75.7 ^b^	74.9 ^b^	73.3 ^b^	0.39	0.033
36–42 day (finisher)						
Dry matter	81.5	84.4	87.3	82.1	0.77	0.165
Crude protein	76.1	79.3	77.3	76.5	0.59	0.283
Ether extract	74.2 ^a^	65.6 ^b^	67.3 ^b^	71.2 ^a,b^	0.61	0.026
Organic matter	81.0	83.1	82.8	83.7	0.43	0.317
Gross energy	82.3	80.6	78.9	71.6	0.52	0.119

^1^ Data represent the mean of 5 cages (10 broiler chicken/cage) per treatment; ^2^ Control—diet without oilseeds; CAM—diet with 15% camelina seeds, FLA—with 15% flax seeds; SUN—diet with 15% sunflower seeds; ^3^ SEM—standard error of the mean; ^4^ *p* < 0.05—statistical differences; ^a,b^—statistical differences.

**Table 5:** Proximate composition, energy value, and mineral contents of meat obtained from broiler chickens fed a control, camelina, flax, and sunflower diet ^1^.

Items	Treatments ^2^				Statistical Parameters	
	Control	CAM	FLA	SUN	SEM ^3^	*p*-Value ^4^
Breast muscle						
Basic nutrients, g/100 g						
Moisture	75.5	76.6	76.3	77.3	0.43	0.146
Protein ^5^	21.4	21.2	21.0	20.3	0.12	0.267
Fat	1.34 ^a^	1.08 ^b^	1.04 ^b^	1.07 ^b^	0.03	0.024
Ash	1.19 ^a^	1.12 ^b^	1.11 ^b^	1.10 ^b^	0.05	0.038
Energy, kcal	97.7	94.5	93.4	90.8	3.18	0.098
Energy, kJ	23.3	22.6	22.3	21.7	0.45	0.098
Mineral elements, mg/kg						
Ca	29.0	30.1	31.5	29.8	0.39	0.184
Mg	15.9	16.5	16.1	16.5	0.13	0.263
P	238	257	245	244	15.85	0.078
Cu	0.046	0.049	0.051	0.043	0.02	0.145
Fe	0.486	0.531	0.496	0.499	0.04	0.209
Zn	0.492	0.512	0.509	0.526	0.03	0.148
Drumstick muscle						
Basic nutrients, g/100 g						
Moisture	73.9	75.6	75.1	76.1	0.09	0.218
Protein ^5^	17.6	18.3	17.8	18.6	0.52	0.123
Fat ^6^	7.50 ^a^	5.10 ^b^	5.06 ^b,c^	4.61 ^c^	0.06	0.037
Ash	1.03	1.01	1.02	1.02	0.08	0.144
Energy, kcal	138 ^a^	119 ^b^	117 ^b^	117 ^b^	5.7	0.042
Energy, kJ	32.9 ^a^	28.5 ^b^	27.9 ^b^	27.9 ^b^	0.89	0.042
Mineral elements, mg/kg						
Ca	7.98	7.69	8.09	7.76	0.09	0.137
Mg	22.2	22.2	22.6	22.1	0.41	0.254
P	194	215	208	199	3.48	0.143
Cu	0.082	0.094	0.091	0.087	0.03	0.095
Fe	0.637 ^b^	0.678 ^a,b^	0.706 ^a^	0.657 ^a,b^	0.01	0.038
Zn	1.53	1.52	1.55	1.54	0.04	0.216

^1^ Data represent the mean of 5 cages (2 broiler chicken/cage) per treatment; ^2^ Control—diet without oilseeds. CAM—diet with 15% camelina seeds, FLA—with 15% flax seeds, SUN—diet with 15% sunflower seeds; ^3^ SEM—standard error of the mean; ^4^ *p* < 0.05—statistical differences; ^a,b,c^—statistical differences; ^5^ Calculated by Kjeldhal nitrogen N × 6.25 (crude protein); ^6^ ether extract—determined with the Soxhlet method.

**Table 6:** Correlation coefficients between basic nutrients and mineral elements in the camelina, flax, and sunflower seeds and the breast and drumstick muscles of broiler chickens fed a control, camelina, flax, and sunflower diet (r) ^1^.

Breast Muscles											Drumstick Muscles									
Items	Ca	Mg	P	Cu	Fe	Zn	Dry Matter	Crude Protein	Fat	Crude Ash	Ca	Mg	P	Cu	Fe	Zn	Dry Matter	Crude Protein	Fat	Crude Ash
Camelina seeds																				
Ca	ns	ns	ns	ns	ns	ns	ns	ns	0.61	ns	ns	ns	ns	ns	ns	ns	ns	ns	0.77	ns
Mg	−0.89	ns	ns	ns	ns	ns	−0.66	−0.71	ns	ns	ns	0.89	ns	ns	ns	0.63	0.62	−0.71	ns	ns
P	0.77	−0.63	−0.85	0.78	0.84	0.71	−0.67	ns	−0.78	−0.76	0.87	−0.73	ns	ns	0.65	0.75	−0.73	ns	−0.73	0.75
Cu	0.75	−0.81	−0.87	0.87	ns	−0.63	ns	ns	−0.66	ns	0.73	−0.86	−0.67	0.85	ns	ns	ns	ns	−ns	ns
Fe	−0.78	ns	−0.73	ns	−0.76	0.67	0.68	−0.76	−0.82	ns	ns	ns	−0.78	−0.77	ns	0.69	0.65	−0.76	−0.82	0.64
Zn	0.66	ns	0.84	−0.92	0.73	ns	0.71	ns	0.74	ns	0.76	0.64	0.85	−0.62	0.73	ns	0.81	ns	0.74	ns
Dry matter	ns	ns	ns	ns	ns	ns	ns	−0.94	ns	ns	ns	ns	ns	ns	ns	ns	ns	−0.94	ns	0.88
Crude protein	ns	ns	ns	ns	ns	ns	ns	ns	ns	ns	ns	ns	ns	ns	ns	ns	ns	ns	ns	ns
Fat	−0.80	−0.63	ns	ns	0.64	−0.87	−0.62	−0.76	−0.81	ns	ns	−0.61	0.89	0.81	0.71	ns	−0.62	−0.76	ns	ns
Crude ash	0.69	ns	0.82	−0.92	ns	ns	ns	ns	0.65	ns	0.65	ns	0.77	−0.92	ns	ns	ns	ns	0.69	0.73
Flax seeds																				
Ca	−0.65	−0.66	ns	0.51	ns	ns	−0.75	−0.81	ns	ns	−0.85	ns	ns	0.66	ns	ns	−0.85	−0.81	ns	ns
Mg	0.67	0.62	ns	ns	ns	ns	0.89	0.73	0.63	ns	0.67	0.65	ns	ns	0.56	ns	0.81	0.73	ns	ns
P	0.75	−0.87	0.63	−0.67	−0.87	0.69	0.83	ns	0.68	0.73	0.66	0.77	0.83	0.87	0.73	0.89	0.73	ns	0.78	0.63
Cu	−0.88	−0.67	−0.87	0.75	0.66	−0.63	ns	−0.63	ns	ns	−0.78	ns	−0.77	−0.75	0.64	0.65	ns	−0.63	ns	ns
Fe	0.74	0.80	ns	ns	0.64	ns	0.83	0.60	0.72	0.68	ns	0.83	ns	ns	0.74	ns	ns	0.60	0.66	0.78
Zn	−0.84	ns	0.75	ns	0.79	−0.87	−0.78	ns	ns	−0.75	−0.81	ns	ns	ns	ns	ns	−0.63	ns	ns	−0.71
Dry matter	0.61	ns	ns	ns	0.68	ns	ns	ns	ns	ns	0.61	ns	ns	ns	0.73	ns	ns	ns	ns	ns
Crude protein	ns	ns	ns	ns	ns	ns	ns	0.74	ns	ns	ns	ns	ns	ns	0.64	0.67	ns	0.74	ns	ns
Fat	−ns	0.91	ns	0.65	ns	−0.65	ns	ns	−0.74	0.84	−ns	0.86	0.62	0.83	ns	−0.61	ns	ns	−0.84	ns
Crude ash	ns	0.73	ns	ns	ns	0.76	0.86	0.69	0.68	ns	ns	0.64	ns	0.84	ns	0.72	0.71	0.69	−0.63	ns
Sunflower seeds																				
Ca	−0.66	−0.65	ns	−0.61	ns	ns	−0.74	−0.83	−0.66	ns	0.86	0.75	ns	−0.65	ns	ns	ns	−0.73	0.76	ns
Mg	0.61	0.69	ns	ns	ns	ns	0.89	0.73	0.63	0.81	0.77	0.64	ns	ns	ns	ns	0.69	0.65	0.85	ns
P	0.75	0.87	0.63	−0.87	−0.83	0.69	0.83	ns	0.78	0.75	0.85	0.67	0.65	−0.83	ns	0.67	0.73	ns	0.76	0.64
Cu	−0.78	−0.67	−0.87	0.75	0.76	−0.90	ns	−0.63	ns	ns	−0.68	−0.61	ns	0.65	0.86	−0.77	ns	−0.73	ns	ns
Fe	0.73	0.8	ns	ns	−0.64	ns	0.80	0.60	0.72	0.62	0.73	−0.89	ns	ns	0.67	ns	0.83	0.82	−0.72	0.72
Zn	−0.82	−0.84	−0.65	0.67	0.73	−0.87	−0.73	−0.61	ns	−0.71	ns	0.74	−0.75	0.77	0.83	−0.67	−0.63	−0.73	ns	−0.81
Dry matter	−0.60	ns	ns	ns	0.78	ns	ns	ns	ns	ns	0.66	ns	ns	ns	ns	ns	ns	ns	ns	ns
Crude protein	ns	0.52	ns	ns	ns	ns	ns	0.74	ns	ns	ns	ns	ns	0.64	ns	ns	ns	0.69	ns	ns
Fat	−0.66	−0.91	0.84	0.65	0.87	−0.65	ns	ns	−0.74	ns	ns	−0.81	0.74	0.85	0.73	0.85	ns	ns	0.71	0.68
Crude ash	0.67	0.73	ns	ns	ns	ns	0.84	0.61	0.81	0.63	0.61	0.76	0.86	0.84	ns	ns	ns	−0.64	0.68	0.71

^1^ significance values *p* < 0.05.

**Table 7:** Effect of dietary inclusion of full-fat seeds on hematological indices and content of plasma elements in broiler chickens fed a control, camelina, flax, and sunflower diet ^1^.

Items	Treatments ^2^				Statistical Parameters	
	Control	CAM	FLA	SUN	SEM ^3^	*p*-Value ^4^
Hematologic indices ^5^						
RBC, 10^12^·L^−1^	2.88	2.92	2.89	3.02	0.05	0.798
HGB, mmol·L^−1^	8.03 ^a^	7.86 ^a^	7.28 ^b^	7.97 ^a^	0.17	0.040
MCHC, mmol·L^−1^	24.3	23.4	22.7	23.5	0.27	0.214
MCH, pg	44.9	43.4	40.7	42.4	0.69	0.144
MCV, fl	115	115	111	112	0.86	0.310
PCV, l·L^−1^	0.330	0.336	0.319	0.340	0.01	0.566
Plasma elements						
Ca, mmol·L^−1^	2.34	2.06	2.07	2.07	0.07	0.455
Mg, mmol·L^−1^	0.843 ^a,b^	0.777 ^b^	0.853 ^a^	0.813 ^a,b^	0.01	0.048
P, mmol·L^−1^	1.97	1.90	2.11	2.13	0.05	0.357
Cu, µmol·L^−1^	4.50	5.30	5.37	4.78	0.25	0.639
Fe, µmol·L^−1^	14.0 ^c^	14.9 ^c^	23.9 ^a^	19.4 ^b^	1.06	<0.001
Zn, µmol·L^−1^	18.8	23.1	24.3	22.5	0.84	0.084

^1^ Data represent the mean of 5 cages (2 broiler chicken/cage) per treatment; ^2^ Control—diet without oilseeds. CAM—diet with 15% camelina seeds. FLA—with 15% flax seeds. SUN—diet with 15% sunflower seeds; ^3^ SEM—standard error of the mean; ^4^ *p* < 0.05—statistical differences; ^a,b,c^—statistical differences; ^5^ RBC—red blood cell, HGB—hemoglobin, MCHC—mean corpuscular hemoglobin concentration, MCH—mean corpuscular hemoglobin, MCV—mean corpuscular volume, PCV—packed cell volume.
